# Randomised active controlled trial examining effects of aerobic exercise, cognitive and music interventions on depression, balance and mobility in schizophrenia

**DOI:** 10.1038/s41598-025-05024-x

**Published:** 2025-06-06

**Authors:** Razieh Khanmohammadi, Hasan Mirali, Hasan Mohammadzadeh, Safiye Ebrahimi, Ina Shaw, Brandon S. Shaw

**Affiliations:** 1 Department of Motor Behavior and Sport Management, Faculty of Sports Sciences, Urmia University, Urmia City, West Azarbaijan Province Iran; 2 School of Sport, Rehabilitation and Exercise Sciences, University of Essex, Wivenhoe Park, Colchester, Essex UK; 3 Division of Public Health, University of the Free State, Bloemfontein, 9300 South Africa

**Keywords:** Cognition, Complementary therapy, Functional capacity, Neuropsychiatric disorders, Psychopathology, Schizophrenia, Physiology, Health care, Signs and symptoms, Diseases, Neurological disorders

## Abstract

Schizophrenia significantly impairs daily functioning, requiring innovative, cost-effective treatments beyond standard antipsychotics, and cognitive interventions. This study examined the individual and combined effects of cognitive, music, and aerobic exercise interventions on depression, balance, and mobility in patients with schizophrenia and severe depression. Eighty-four male patients with schizophrenia and severe depression from an inpatient psychiatric centre participated in a 12-week, single-blind, randomised active-controlled trial. Participants were systematically assigned to one of seven equal groups (*n* = 12 each): aerobic exercise (AerG), cognitive rehabilitation/treatment-as-usual (CogG), music intervention (MusG), aerobic exercise + music intervention (A&MG), aerobic exercise + cognitive intervention (A&CG), cognitive intervention + music intervention (C&MG), and a comprehensive combination of all three modalities (ACMG). Each intervention was delivered over 60 min, three times weekly for 12 weeks. The study employed the Beck Depression Inventory Short Form, Stork Balance Test, and modified Timed Up and Go Test to assess improvements in depression, balance, and mobility. Statistical analyses were conducted using paired t-tests for within-group comparisons and ANCOVA with Bonferroni post hoc tests for between-group differences, with significance set at *p* ≤ 0.05. Results showed significant improvements in depression, balance, and mobility across all treatment groups. The CogG group outperformed both AerG and MusG in all outcomes, establishing it as the gold-standard comparator. A&CG yielded greater benefits than other single or dual-modality groups, while the multimodal ACMG group demonstrated the most substantial improvements across all measures. These findings highlight the practical value of incorporating multimodal interventions into standard care to improve both mental health and physical function, offering a scalable, cost-effective approach to addressing the diverse needs of this population of patients with schizophrenia and severe depression. Implementing such interventions in psychiatric care settings could lead to more comprehensive and effective treatment strategies for improving patient outcomes.

Schizophrenia is a long-term mental health condition resulting from a combination of genetic and environmental factors, characterised by chronic psychological symptoms. The World Health Organisation (WHO) reports that over 26 million individuals worldwide are affected by schizophrenia. In Iran, the prevalence was reported at 17% in 2001^1^ while a 2014 adult psychiatric morbidity survey (APMS) found that approximately 0.5% of individuals aged 16 years or older in England had received a diagnosis of a psychotic disorder, including schizophrenia^2^. These statistics indicate that schizophrenia’s prevalence is rising, making it a significant public health concern^3^.

Emotional disturbances are common in individuals with schizophrenia, with depression affecting 22–75% of patients, depending on the diagnostic criteria used^4,5^. This depression leads to increased recurrence or worsening of the disorder, drug-related issues, decreased life satisfaction, mental dysfunction, poor social relationships, and heightened risks of violence, arrest, victimisation, and suicide, which is a leading cause of death among these individuals. Additionally, the depression associated with schizophrenia is linked to cognitive impairments, such as decreased attention and reduced executive functions^6^. Therefore, treatment options must address the effects of depression and cognitive function to provide comprehensive care for individuals with schizophrenia^5^. Research is needed especially in patients with schizophrenia and severe depression because this population faces significant challenges, including impaired mobility, poor balance, and when combined with their depressive symptoms, meaningly contribute to reduced independence and quality of life.

The most debilitating issue associated with schizophrenia is impairments in everyday functioning, which affect life, physical, and health maintenance^7^. The condition is also linked to a high incidence of neurological symptoms and ongoing psychomotor dysfunction^8^. Research shows that schizophrenia can lead to increased body sway due to structural disorders in the cerebellum, affecting balance, stability, and mobility^9^. Additionally, medications prescribed for schizophrenia can alter brain areas like the basal ganglia, crucial for postural stability^10,11^. These impairments suggest accelerated aging in schizophrenia^12^, which, when combined with low physical activity, leads to poor functional capacity and higher rates of falls, hospitalisations, and mortality^13^. Furthermore, antipsychotic drugs increase the risk of comorbidities, such as reduced bone mass and fractures^14^.

Typical treatments for schizophrenia include antipsychotic medications and cognitive behavioural interventions. However, randomised trials on the efficacy of antidepressants for treating depression in this population show limited results^15^. This has led to the advocacy for a combination of treatments, particularly non-pharmacological interventions, to address the cluster of symptoms associated with schizophrenia^16^. Additionally, rising costs of pharmacological treatments and limited financial resources in both low- and middle-income countries (LMICs) like Iran and high-income countries like the UK highlight the urgent need for cost-effective and scalable multimodal treatment options that address multiple symptoms and consequences of schizophrenia^17,18^.

In addition to evaluating cost-effective treatments that improve critical symptom areas, there is a need to assess novel treatment methods, both in isolation and in combination with proven therapies^19^. Aerobic exercise has demonstrated benefits for schizophrenia, improving symptoms like depression^19^ and enhancing physical performance, such as gait and balance^20^. Cognitive interventions have been shown to enhance cognitive function through neuroplasticity, reduce depressive symptoms^21^, and improve physical outcomes like balance and walking speed, particularly in the elderly^22^. Additionally, music interventions may serve as a valuable complementary treatment, providing a non-verbal medium for emotional expression, fostering social interaction, stimulating cognitive functions, and managing specific symptoms like auditory hallucinations and disorganised thinking by redirecting attention from distressing experiences^22^.

Aerobic exercise, cognitive, and music interventions each improve depression, balance, and mobility through distinct but complementary mechanisms. Aerobic exercise enhances neuroplasticity, and increases the availability of neurotransmitters such as serotonin and dopamine, which are critical in mood regulation and motor control^23^. It also improves cardiorespiratory fitness, muscle strength, proprioception, and sensorimotor integration, all of which contribute to better balance and mobility^24^. Cognitive interventions targets dysfunctional thought patterns, and promotes cognitive restructuring, which not only alleviates depressive symptoms, but also enhances executive functions like attention, and planning^25^. Improved executive function is crucial for postural control, and safe mobility, particularly in individuals with schizophrenia who often suffer from cognitive deficits^26^. Meanwhile, music provides a safe space for emotional expression and can enhance mood-stabilising neurochemistry^27^. Music-based rhythmic stimulation has also been shown to improve gait, and balance by enhancing auditory-motor synchronisation, and movement timing^28^. Music-based training not only improves motor performance, but also enhances perceptual timing, and adaptability to temporal changes^29^.

Our conceptual framework defines a successful endpoint in the treatment of schizophrenia as adequate psychological and physical functioning in an in-patient setting. However, this has been a highly neglected topic in the past^30–32^. Given the challenges posed by schizophrenia, and especially when combined with severe depression, a comprehensive multimodal treatment approach is crucial. While drug therapies come with potential side effects, exploring multimodal drug and non-drug intervention strategies is essential to enhance effectiveness and provide complementary benefits. In this context, this study’s objective was to investigate and compare the individual and combined effects of cognitive, music, and aerobic exercise therapies on depression, balance, and mobility in patients with schizophrenia and severe depression.

## Materials and methods

### Trial design

**Fig. 1:**
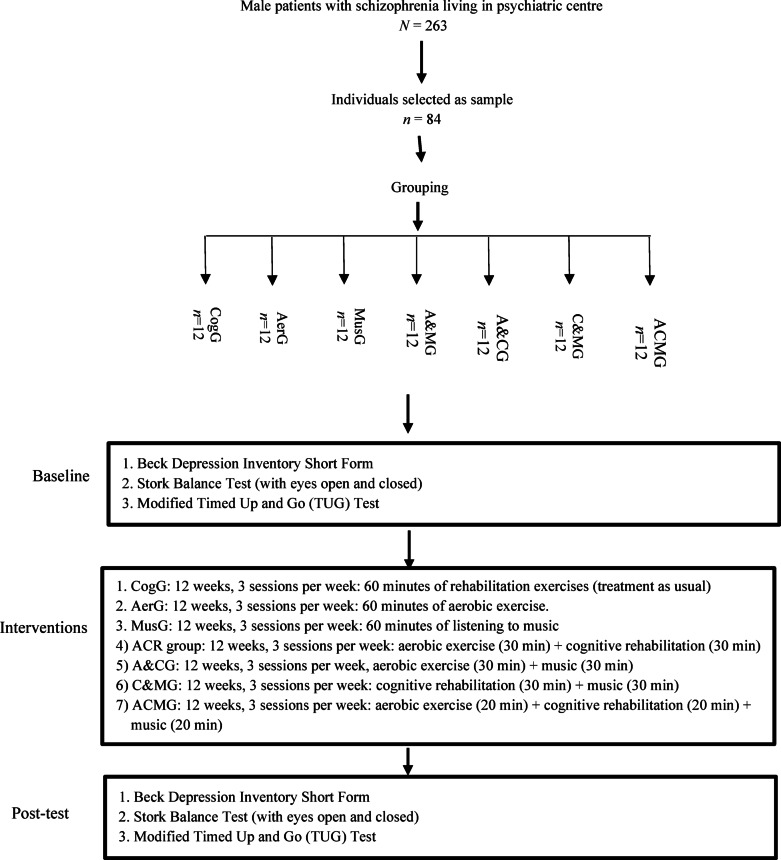
Flow chart of study design. CogG: cognitive rehabilitation/treatment-as-usual group; AerG: aerobic exercise group; MusG: music intervention group; aerobic exercise + music intervention group (A&MG), A&CG: aerobic exercise + cognitive rehabilitation group; C&MG: cognitive rehabilitation + music intervention group; ACMG: aerobic exercise + cognitive rehabilitation + music intervention group.

This study utilised a 12-week single-blinded, randomised active-controlled seven-arm trial (RAT) conducted in the in-patient Psychiatric Centre of Urmia, Iran. Eighty-four male adults with diagnosed schizophrenia were recruited and evaluated for eligibility based on medical records. A male-only sample was selected based on the demographic composition of the inpatient psychiatric centre, which housed only male patients during the recruitment period due to religious and cultural considerations. This approach also allowed for greater control over sex-related confounding variables such as hormonal and physiological differences affecting depression, balance, and mobility, with future studies planned to include female participants to enhance generalisability through a stepwise research design. Participants were randomly allocated by the project coordinator using random allocation software to one of seven groups of equal numbers (*n* = 12). Each cohort comprised 12 patients who were blinded to the study protocol. The groups were an aerobic exercise (AerG), cognitive intervention (CogG), music intervention (MusG), aerobic exercise + music intervention (A&MG), aerobic exercise + cognitive intervention (A&CG), cognitive intervention + music intervention (C&MG), and aerobic exercise + cognitive intervention + music intervention (ACMG) group. This study followed the CONSORT guidelines (Fig. 1). A 12-week intervention duration was selected based on prior research indicating that this timeframe is sufficient to elicit clinically meaningful changes in both psychological and physical outcomes in individuals with schizophrenia^33–35^. Studies have shown that interventions such as aerobic exercise, cognitive rehabilitation, and music interventions typically begin to produce measurable improvements in depression, balance, and mobility within 10 to 12 weeks^33^. In addition, a 12-week clinical intervention was chosen to balance the need for sufficient observation time with practical considerations and statistical power. In this regard, 12 weeks provided a sufficiently long period to observe changes or improvements in participants’ conditions or behaviours without being so long that it becomes too resource-intensive and impractical for real-world applications. In addition, a 12-week period allowed for meaningful changes in health outcomes, making the results more clinically relevant.

This study employed a randomized active-controlled trial (RAT) instead of a traditional randomized controlled trial (RCT) for several reasons. Primarily, it aimed to compare new interventions against the most effective existing treatment, that of cognitive rehabilitation or treatment-as-usual^36^. This approach ensured adherence to the ethical and legal standards of care by providing all inpatients with an established treatment, thereby avoiding the ethical dilemma of withholding effective care or exposing patients to unnecessary risks. Additionally, using an RAT maximized the benefits for all participants, ensuring they received a potentially effective intervention rather than a placebo or inactive control^37^. This design was in consideration with the World Medical Association’s Declaration of Helsinki, as revised in 2013, which states that “the benefits, risks, burdens, and effectiveness of a new intervention must be tested against those of the best proven intervention(s)”^38^ which in the case of treatment for schizophrenia is a cognitive intervention. This study received ethical approval on 20/09/2023 from the Biomedical Research Ethics Committee of the Institute of Physical Education and Sports Sciences, Urmia University (ID IR.SSRC.REC.1402.136). Final post-testing for this study took place 19/01/2024, and the study was also retrospectively registered as a clinical trial on the Iranian Registry of Clinical Trials (https://irct.behdasht.gov.ir/) (IRCT20190908044722N2) on 15/01/2024.

### Participants

The study population comprised 263 male patients diagnosed with schizophrenia and high levels of depression (i.e., BDI-SF scores of 16–39), residing in the in-patient Psychiatric Centre of Urmia, Iran. A preliminary power analysis was used to avoid a type II error. Using a power analysis, the minimum sample size was determined to be 60 patients using G*power software version 3.1.9.2 (Effect size F: 0/7; α err prob: 0/05; power: 0/95; numerator: 10; number of groups: 7; number of covariate: 1). However, to allow for potential dropouts, a total of 84 participants (average age 39.12 ± 8.90 years) were selected for the study.

Inclusion criteria were (a) ability to complete and sign an informed consent, (b) at least one year of hospitalisation, (c) stable medication and diet, (d) ability to perform aerobic exercises, (e) a score above 11 in the Beck Depression Inventory Short Form (BDI-SF), (f) absence of cardiovascular diseases, glandular diseases, or other physical disorders, and (g) no structured physical activity regime as defined by less than one hour of physical activity per week. A BDI-SF score of ≥ 11 was selected as this specific cutoff was selected since a BDI-SF score of ≥ 11 has been recommended to balance sensitivity and specificity when screening for moderate depression^39,40^. A score of 11 or higher has also been used in clinical and research settings as a conservative threshold to identify individuals with meaningful depressive symptoms, distinguishing them from those with transient or mild symptoms^39^. Exclusion criteria were (a) severe symptoms of schizophrenia requiring hospitalisation, (b) unwilling to participate in exercise, (c) history of intellectual disabilities or dementia, (d) presence of physical disabilities or other diseases, (e) participation in another study, and (f) participants who were likely to miss more than three intervention sessions or expressed an unwillingness to adhere to the full 12-week protocol, as this could compromise data integrity and intervention consistency. Table 1 presents the participant descriptive data for these participants.

**Table 1:** Participant baseline characteristic data.

Group	AerG (*n* = 12)	CogG (*n* = 12)	MusG (*n* = 12)	A&MG (*n* = 12)	A&CG (*n* = 12)	C&MG (*n* = 12)	ACMG (*n* = 12)
Age (years)	35.33 ± 10.50	36.25 ± 7.21	37.25 ± 9.92	42.08 ± 8.45	39.08 ± 8.49	42.00 ± 8.20	41.83 ± 8.44
BDI-SF score	33.33 ± 2.99	33.92 ± 2.50	31.58 ± 2.81	32.92 ± 1.97	31.25 ± 3.25	31.58 ± 3.20	35.58 ± 2.43

*:significant difference *p* ≤ 0.05; Data are presented as means ± SD; AerG: aerobic exercise group; CogG: cognitive rehabilitation group; MusG: music intervention group; A&MG: aerobic exercise + music intervention group; A&CG: aerobic exercise + cognitive rehabilitation group; C&MG: cognitive rehabilitation + music intervention group; ACMG: aerobic exercise + cognitive rehabilitation + music intervention group; BDI-SF: Beck Depression Inventory Short Form.

### Outcome assessments

The Beck Depression Inventory Short Form (BDI-SF) was utilised to assess depression in this study^41^. This is a self-report inventory consisting of 13 multiple-choice questions designed to measure the severity of depression. Each question has four possible responses, ranging in intensity from 0 (do not feel sad) to 3 (I am so sad or unhappy that I can’t stand it). The BDI-SF score interpretation demonstrates that a score of 0–4 indicates minimal or no depression, 5–7 suggests mild depression, 8–15 reflects moderate depression, and 16–39 signifies severe depression.

The Stork Balance Test was performed in both eyes open and eyes closed conditions using each participant’s preferred leg^42^. The participants were asked to stand on one leg (bare feet) while the hands were in front of the chest. The alternate leg was then raised up to a height of at least 5 centimetres (cm) above the ground. At this point, a stopwatch was started and stopped when each patient was not able to keep the original position. The time to maintain the balance was recorded, with a maximum score of 120 s.

The modified Timed Up and Go (TUG) Test was utilised to measure balance and mobility in this study^43^. In this assessment, each participant began by sitting on a chair with a height of 46 cm and a handle height of 63 cm. Once hearing the ‘go’ command, participants were required to stand and walk a 3 m (m) path. At the end of the 3 m path, each participant was to turn around and return to the chair and retake their seat. The fastest time of two attempts was recorded and analysed.

### Interventions

The CogG, A&CG, C&MG, and ACMG participated in the proven “gold-standard”, and comparator control treatment of cognitive rehabilitation using the Attentive Rehabilitation of Attention and Memory (ARAM) programme^44^ three times weekly for 12 weeks. The ARAM programme is a cognitive rehabilitation tool based on the attention hierarchical model and Baddeley’s working memory model, aimed at improving the various dimensions of attention (selective, sustained, transitive, and divided) and memory^44^. Patients were seated at a personal computer (HP, i5 quad core desktop, Palo Alto, California) preloaded with the ARAM computerised cognitive rehabilitation software programme and completed 10 graded progressive tasks. The software comprises 10 graded progressive tasks, adjusted according to the number of two-sided stimuli, their presentation speed, complexity, and changing rules. The programme’s principles include: (1) hierarchical task organisation with increasing difficulty based on user feedback, (2) immediate rewards for correct task completion, (3) task design based on different dimensions of attention, (4) engaging tasks to enhance patient motivation, (5) task repetition until the patient achieves a suitable level, and (6) patient performance-based progression without the need for therapist intervention to advance the task level. Each ARAM session lasted approximately 45 min.

The AerG, A&MG, A&CG and ACMG performed aerobic exercise according to the American College of Sports Medicine (ACSM) physical activity recommendations^45^. Each session began with a 10-minute warm-up using a cycle or treadmill (selected according to each patient’s preference and abilities), this was followed by 45 min of aerobic exercise, and concluded with a 5-minute cool-down, all under the supervision of a qualified trainer. Each participant’s exercise intensity was set at 60-70% of the maximum heart rate (HRmax) for the first three weeks, and ~ 75% HRmax for weeks 4–12. Predicted maximum heart rate was calculated as follows: 220 − age. Heart rate was monitored throughout the exercises using a Beurer Model PM15 heart rate monitor (Beurer GmbH, Ulm, Germany).

The MusG, A&MG, C&MG, and ACMG participated in thrice-weekly music interventions. The music intervention was delivered within a structured music therapy framework based on the clinical practice model^46^, a well-established theoretical approach in music therapy that emphasises the use of music to support emotional, psychological, and physiological well-being in individuals with cognitive impairments. This model advocates for personalised and patient-centered interventions, utilizing music to music to reduce anxiety, alleviate stress, and enhance relaxation-objectives that align with the therapeutic goals of our study. Although active participation methods hold significant value, they were considered less feasible for this patient group due to the cognitive challenges these individuals face, including limited capacity for engagement. As a result, our intervention primarily employed a receptive music therapy approach. This method involved passive listening to music selected based on individual preferences, as determined through questionnaires and interviews. The style of music varied between patients and sessions, with an emphasis on choosing music related to personal experiences. The researcher prepared a list of suggested music genres (e.g., classical, jazz, and world music) and allowed patients to select tracks^47^. During individual sessions, each standard music track, lasting 20 min, was divided into several phases to gradually guide the patient into relaxation using the ‘U sequence’ method^47^. This method involved reducing the musical rhythm, orchestral formation, frequency, and volume (downward U-phase), followed by a recovery phase (upward ‘U’ segment). Music was delivered to each patient via personal headphones at a volume selected by the participant. The sessions were facilitated by a licensed, and experienced music therapist with formal training in music therapy, holding a PhD, and extensive clinical experience in delivering evidence-based interventions tailored for neurological, and psychiatric populations. The therapist employed their professional expertise to select and guide patients through the listening protocol.

### Statistical methods

Data are presented as mean ± standard deviation (SD) unless stated otherwise. The analytical design was formulated to ensure robust and accurate statistical evaluation. First, the normality of data distribution was assessed using the Shapiro-Wilk test, while the homogeneity of variances was verified using Levene’s F test. Additionally, the homogeneity of regression slopes was examined using analysis of covariance (ANCOVA) to ensure the appropriateness of the chosen statistical models. For intragroup comparisons, paired t-tests were employed to evaluate the effects of the intervention on depression, cognitive function, balance, and mobility within each group. To compare these variables between groups, an ANCOVA was conducted, with the pre-test scores of the research variables included as covariates to control for baseline differences. Bonferroni’s post hoc test was applied for multiple comparisons to adjust for Type I error inflation due to the simultaneous use of multiple statistical tests. Statistical analyses also included effect size calculations (Cohen’s d) to verify the magnitude of the result of the interventions on the analyzed variables, and were classified as “small” (< 0.2), “medium” (0.2 to 0.8), and “large” (> 0.8). The nominal level of significance was set at alpha ≤ 0.05, and all statistical analyses were performed using the Statistical Package for the Social Sciences (SPSS) version 26.0 (IBM, New York, USA).

## Results

A total of 84 patients with schizophrenia with severe depression (i.e., BDI-SF scores of 16–39) participated in this study (32% of the total population, and average age 39.12 ± 8.90 years). No adverse effects were reported as a consequence of the study. There were no statistically significant (*p* > 0.05) differences in baseline characteristics between groups (Table 1). The results of Shapiro-Wilk, Levin’s F, and ANCOVA tests showed that when considering that the calculated significance value was more than five hundredths, the assumption of normality of data distribution, homogeneity of variances and homogeneity of regression slope were met.

### Within-group comparisons

Table 2 presents the results of the paired t-tests conducted across all treatment groups, evaluating changes in BDI-SF scores, Stork Balance Test (eyes open and closed), and TUG Test from baseline to post-test. Significant improvements were observed in all groups, including those receiving single-modality treatments (AerG, CogG, MusG) and those undergoing combined or multimodal interventions (A&MG, A&CG, C&MG, ACMG). The statistical analyses demonstrate consistent and meaningful significant changes across all outcome measures.

**Table 2:** Effects of 12 weeks of aerobic exercise, cognitive, and/or music intervention on depression, cognitive impairment, balance, and mobility in male patients with schizophrenia.

Group	Variable	Baseline	Post-test	*P*-value	Post-hoc
AerG (*n* = 12)	BDI-SF score	33.33 ± 2.99	30.80 ± 2.04*	0.001	
	Stork Balance Test (eyes open) (seconds)	5.04 ± 1.89	6.67 ± 2.12*	0.001	
	Stork Balance Test (eyes closed) (seconds)	3.34 ± 2.37	4.49 ± 2.07*	0.001	
	TUG Test (seconds)	22.37 ± 1.76	20.85 ± 2.13*	0.019	
CogG† (*n* = 12)	BDI-SF score	33.92 ± 2.50	26.82 ± 3.68*	0.001	AerG: 0.003, MusG: 0.021
	Stork Balance Test (eyes open) (seconds)	4.54 ± 2.55	9.82 ± 2.58*	0.001	AerG: 0.001, MusG: 0.001
	Stork Balance Test (eyes closed) (seconds)	2.89 ± 1.41	6.22 ± 1.44*	0.001	AerG: 0.032, MusG: 0.005
	TUG Test (seconds)	21.75 ± 1.49	18.16 ± 2.54*	0.001	AerG: 0.018 MusG: 0.011
MusG (*n* = 12)	BDI-SF score	31.58 ± 2.81	29.54 ± 4.03*	0.039	
	Stork Balance Test (eyes open) (seconds)	6.43 ± 3.28	7.82 ± 3.30*	0.001	
	Stork Balance Test (eyes closed) (seconds)	3.10 ± 1.70	4.35 ± 1.07*	0.001	
	TUG Test (seconds)	22.48 ± 0.93	21.04 ± 1.84*	0.001	
A&MG (*n* = 12)	BDI-SF score	32.92 ± 1.97	25.82 ± 2.96*	0.001	
	Stork Balance Test (eyes open) (seconds)	5.24 ± 2.25	9.55 ± 2.26*	0.001	
	Stork Balance Test (eyes closed) (seconds)	2.94 ± 1.43	5.59 ± 1.31*	0.001	
	TUG Test (seconds)	19.35 ± 1.86	17.14 ± 1.16*	0.001	
A&CG^††^ (*n* = 12)	BDI-SF score	31.25 ± 3.25	21.18 ± 2.56*	0.001	AerG: 0.001, CogG: 0.011, MusG: 0.001
	Stork Balance Test (eyes open) (seconds)	4.13 ± 2.72	11.47 ± 3.00*	0.001	AerG: 0.001, CogG: 0.036, MusG: 0.001
	Stork Balance Test (eyes closed) (seconds)	2.17 ± 0.96	7.44 ± 1.02*	0.001	AerG: 0.001, CogG: 0.038, MusG: 0.001
	TUG Test (seconds)	18.94 ± 0.94	14.27 ± 2.47*	0.001	AerG: 0.001, CogG: 0.005, MusG: 0.001
C&MG (*n* = 12)	BDI-SF score	31.58 ± 3.20	25.54 ± 2.25*	0.001	
	Stork Balance Test (eyes open) (seconds)	5.61 ± 3.60	8.91 ± 3.74*	0.001	
	Stork Balance Test (eyes closed) (seconds)	3.88 ± 1.56	6.10 ± 1.68*	0.001	
	TUG Test (seconds)	20.06 ± 2.18	17.71 ± 2.49*	0.004	
ACMG^†††^ (*n* = 12)	BDI-SF score	35.58 ± 2.43	18.42 ± 1.56*	0.001	AerG: 0.001, CogG: 0.0001, MusG: 0.001, A&MG: 0.001, C&MG: 0.002, A&CG: 0.001
	Stork Balance Test (eyes open) (seconds)	3.48 ± 2.42	14.26 ± 2.72*	0.001	AerG: 0.001, CogG: 0.001, MusG: 0.001, A&MG: 0.001, C&MG: 0.001, A&CG: 0.001
	Stork Balance Test (eyes closed) (seconds)	2.48 ± 1.63	11.04 ± 1.97*	0.001	AerG: 0.001, CogG: 0.001, MusG: 0.001, A&MG: 0.001, C&MG: 0.001, A&CG: 0.001
	TUG Test (seconds)	19.39 ± 2.10	11.54 ± 1.41*	0.001	AerG: 0.001, CogG: 0.001, MusG: 0.001, A&MG: 0.001, C&MG: 0.001, A&CG: 0.006

Data are presented as means ± SD; *: significant difference *p* ≤ 0.05 from baseline to post-test; ^†^: Most effective single-modality treatment as determined by analysis of covariance (ANCOVA) with Bonferroni correction; ^††^: Most effective dual-modality treatment as determined by analysis of covariance (ANCOVA) with Bonferroni correction; ^†††^: Most effective treatment overall as determined by analysis of covariance (ANCOVA) with Bonferroni correction; AerG: aerobic exercise group; CogG: cognitive rehabilitation group; MusG: music intervention group; A&MG: aerobic exercise + music intervention group; A&CG: aerobic exercise + cognitive rehabilitation group; C&MG: cognitive rehabilitation + music intervention group; ACMG: aerobic exercise + cognitive rehabilitation + music intervention group; BDI-SF: Beck Depression Inventory Short Form; TUG Test: modified Timed Up and Go Test.

### Main and interactive effects

In Table 3, the main effects of groups, pre-test, and the interaction effects between group and pre-test (Group × Pre-test) were examined. The results showed that for the variables Stork Balance Test (eyes open and closed), the main effect of group (*F*(6,63) = 13.404 and *F*(6,62) = 17.390) and pre-test (*F*(1,63) = 117.959 and *F*(1,62) = 30.601) were significant (*p* < 0.05). However, the interaction effect between group and pre-test was not significant in these variables (*F*(6,63) = 0.532, *p* = 0.782 and *F*(6,62) = 1.553, *p* = 0.176). In the BDI-SF score variable, only the main effect of the pre-test was significant (*F*(1,63) = 16.508, *p* = 0.001), while the main effect of the group (*F*(6,63) = 0.625, *p* = 0.710) and the interaction effect (*F*(6,63) = 1.132, *p* = 0.354) were not significant. In the TUG Test variable, the main effect of the pre-test was significant (*F*(1,63) = 23.398, *p* = 0.001), but the main effect of the group (*F*(6,63) = 1.301, *p* = 0.270) and the interaction effect (*F*(6,63) = 1.795, *p* = 0.115) were not significant. Overall, the pre-test played an important role in predicting outcomes, while the interaction effects did not play a significant role.

**Table 3:** Main and interactive effects of 12 weeks of aerobic exercise, cognitive, and/or music intervention on depression, cognitive impairment, balance, and mobility in male patients with schizophrenia.

Variable	Source	Type III Sum of Squares	df	Mean Square	F	*P*-value
BDI-SF score	Group	24.490	6	4.082	0.625	0/710
	Pre-test	107.798	1	107.798	16.508	0.001^*^
	Group* Pre-test	44.358	6	7.393	1.132	0.354
	Error	411.387	63	6.530		
Stork Balance Test (eyes open) (seconds)	Group	220.643	6	36.774	13.404	0.001^*^
	Pre-test	323.629	1	323.629	117.959	0.001^*^
	Group* Pre-test	8.756	6	1.459	0.532	0.782
	Error	172.845	63	2.744		
Stork Balance Test (eyes closed) (seconds)	Group	151.752	6	25.292	17.390	0.001^*^
	Pre-test	44.505	1	44.505	30.601	0.001^*^
	Group* Pre-test	13.554	6	2.259	1.553	0.176
	Error	90.170	62	1.454		
TUG Test (seconds)	Group	23.690	6	3.948	1.301	0/270
	Pre-test	71.010	1	71.010	23.398	0.001^*^
	Group* Pre-test	32.681	6	5.447	1.795	0.115
	Error	191.200	63	3.035		

*: significant difference *p* ≤ 0.05; BDI-SF: Beck Depression Inventory Short Form; TUG Test: modified Timed Up and Go Test.

### Between-group comparisons

The ANCOVA results and Bonferroni post hoc comparisons (Table 2) revealed significant effects for the BDI-SF score, with the pretest (F (1, 69) = 16.826, *p* = 0.001) showing a moderate effect size (ES = 0.196) and the group (F (6, 69) = 35.019, *p* = 0.001) demonstrating a large effect size (ES = 0.753). For the Stork Balance Test with eyes open, both the pretest (F (1, 69) = 151.353, *p* = 0.001) and group (F (6, 69) = 41.430, *p* = 0.001) were significant, with large effect sizes (ES = 0.687) and (ES = 0.783), respectively. Similarly, in the Stork Balance Test with eyes closed, the pretest (F (1, 69) = 43.178, *p* = 0.001) had a moderate effect size (ES = 0.385), while the group (F (6, 69) = 44.664, *p* = 0.001) exhibited a large effect size (ES = 0.795). The TUG Test showed significant effects for the pretest (F (1, 69) = 29.525, *p* = 0.001) with a moderate effect size (ES = 0.300) and the group (F (6, 69) = 30.740, *p* = 0.001) with a large effect size (ES = 0.728). The ANCOVA results revealed statistically significant differences in post-test scores across all outcome measures, including depression (F(6) = 16.83, *p* = 0.001), balance with eyes open (F(6) = 41.43, *p* = 0.001), balance with eyes closed (F(6) = 44.66, *p* = 0.001), and mobility (F(6) = 30.74, *p* = 0.001).

Bonferroni post hoc tests further detailed these differences that the MusG group had the highest level of depression (*P* ≤ 0.05) and demonstrated the poorest performance in all functional tests (balance and TUG) (*P* ≤ 0.05). In contrast, the ACMG group had the lowest level of depression (*P* ≤ 0.05) and exhibited the best performance in all tests (*P* ≤ 0.05). The AerG group was more depressed compared to the A&CG, A&MG, C&MG, and ACMG groups (*P* ≤ 0.05), but showed no significant difference with the MusG group (*P* ≥ 0.05). In functional tests, this group also performed worse than the CogG, A&CG, A&MG, and ACMG groups (*P* ≤ 0.05), but no significant difference was observed with the MusG group and, in some cases, with the C&MG group (*P* ≥ 0.05). The CogG group was less depressed than the MusG group (*P* ≤ 0.05), but showed higher levels of depression compared to the ACMG group (*P* ≤ 0.05) and had no significant difference with the A&MG and C&MG groups (*P* ≥ 0.05). In functional tests, this group performed worse than the ACMG group and, in some cases, worse than the A&CG and A&MG groups (*P* ≤ 0.05), but no significant difference was observed with the MusG and C&MG groups (*P* ≥ 0.05). The A&CG group was less depressed than the A&MG and C&MG groups (*P* ≤ 0.05), but had higher levels of depression compared to the ACMG group (*P* ≤ 0.05). In functional tests, this group performed worse than the A&MG, C&MG, and ACMG groups (*P* ≤ 0.05), but performed better than the MusG and AerG groups. Finally, no significant difference was observed between the A&MG and C&MG groups in terms of depression and performance across all tests (*P* ≥ 0.05). Overall, the MusG group had the highest level of depression and the poorest performance, while the ACMG group showed the lowest level of depression and the best performance.

The results demonstrated that the CogG group (active control group) had significantly lower levels of depression and better performance in the Stork Balance Test (eyes open), Stork Balance Test (eyes closed), and TUG Test compared to the MusG and AerG groups (*P* ≤ 0.05). However, the CogG group (active control group) exhibited higher levels of depression and weaker performance compared to the A&CG and ACMG groups (*P* ≤ 0.05). Additionally, no significant differences were observed between the CogG group (active control group) and the A&MG and C&MG groups in terms of depression and performance (*P* ≥ 0.05).

## Discussion

This study compared the effects of cognitive, music, and aerobic interventions, both individually and in combination, on depression, balance, and mobility in patients with schizophrenia with severe depression. All interventions led to improvements in depression, balance, and mobility across, but cognitive intervention alone proved superior to aerobic or music intervention when used as a sole modality. Furthermore, combining cognitive and aerobic interventions was more effective than any sole or dual-modality treatment. Notably, the triple-modality intervention (aerobic, cognitive, and music interventions) yielded the greatest improvements across all outcomes. Importantly, no adverse events were reported among participants who completed the treatments.

The finding that the cognitive intervention was the most effective sole modality aligns with its status as the “gold standard” for treating schizophrenia^20,22,30,48^, and it served as the active control in this study. A key insight from this study is that any effective dual- or triple-modality intervention had to include cognitive intervention. Large-scale studies have shown cognitive interventions benefits patients with schizophrenia, especially those not fully responsive to medication^48^, and early intervention may help prevent illness progression by boosting self-confidence and reducing depression^20,22^. Improving cognitive function can enhance balance in patients with schizophrenia, as balance issues are linked to reduced cognitive performance and slower central information processing^49^. Cognitive interventions, whether alone or combined with other modalities, may reduce fall risk by addressing these deficits. Additionally, since cognitive interventions have been shown to alleviate depression, which also increases fall risk^50^, its benefits extend beyond mental health. While this study uniquely highlights improvements in mobility (as measured by the TUG test), previous research has shown cognitive interventions enhance executive functions, aiding daily activities^51^.

This study adds to the evidence that music interventions are a valuable adjunctive treatment for improving depression, balance, and mobility in schizophrenia. Integrating music into clinical settings allows patients to express emotions, reduce stress, and improve mood regulation^52^. It also provides non-verbal communication, aiding those with cognitive and emotional difficulties. Music interventions enhance socialisation, relaxation, and empowerment, while its rhythmic components stimulate neural pathways linked to emotional processing, potentially reducing depressive symptoms^21,53^. Research shows that music interventions can improve physical performance, including balance, in various populations such as stroke patients, those with Parkinson’s disease, the elderly, and healthy individuals^54^. These improvements reduce postural issues and fall risk. Recent studies support the present findings that music interventions enhance mobility, particularly through rhythmic auditory stimulation and movement exercises that target motor control^55^. Regular music intervention sessions improve gait stability, balance, and mobility in schizophrenia^56^, highlighting its potential as a complementary treatment to address both cognitive and physical aspects of the disorder.

Notably, this study successfully reduced high depression scores. The high depression scores observed in this study can be attributed to several factors specific to the participant population and study setting. In this regard, all participants were inpatients at a psychiatric facility, and all individuals displayed with more severe and persistent symptoms, including higher levels of depression, when compared to those in outpatient or community settings^57^. This is because long-term hospitalization, social isolation, and reduced autonomy may further contribute to elevated depressive symptoms^57^. While severe depression (i.e., BDI-SF scores of 16–39) are less common they can occur^58,59^, particularly in chronic cases with significant negative symptoms, poor social support, or co-occurring affective disorders^60^. Additionally, the Beck Depression Inventory Short Form (BDI-SF) used in this study, while a validated measure of depression especially for detecting depression in chronic schizophrenia^61^, it may yield higher scores in populations with cognitive impairments or flattened affect, common in schizophrenia^62^.

Several mechanisms may explain how non-physical activity-based interventions, such as cognitive, and music-based interventions, can improve balance and mobility comparably to or even more than aerobic exercise. Specifically, previous meta-analyses have demonstrated that the effects of traditional exercise on improvement of functional performance in patients with schizophrenia tend to be limited^63^. This is because cognitive interventions that enhance executive function, attention, and sensorimotor integration, are crucial for postural control^63^, and gait adaptation^63^. Similarly, music-based interventions engage rhythm and auditory-motor synchronisation, promoting gait stability and motor coordination^64^. Moreover, these interventions may reduce fall-related anxiety and enhance movement confidence, possibly even more so than the physical activity-based interventions as demonstrated by this study, further supporting mobility improvements^65^. While aerobic exercise directly enhances physical fitness, the superior benefits of cognitive and music interventions likely stem from their impact on neural plasticity, sensorimotor processing, and motor learning, all of which play a fundamental role in balance and mobility^66,67^.

This study demonstrates that integrating aerobic exercise into treatment plans is promising for addressing depression, balance, and mobility challenges in patients with schizophrenia, ultimately enhancing their well-being and treatment outcomes. Aerobic training has emerged as an effective intervention for alleviating depressive symptoms, a common comorbidity that complicates schizophrenia and impairs functioning. It influences mood regulation and cognitive function through neurobiological mechanisms, promoting neuroplastic changes in the brain and upregulating key neurotransmitters like serotonin and dopamine^68^. Additionally, aerobic exercise releases endorphins and neurotrophic factors, which have antidepressant effects and improve emotional resilience^69^. Research indicates that aerobic training significantly reduces depressive symptoms and improves quality of life and functional outcomes, making it an accessible and cost-effective adjunctive treatment for individuals with schizophrenia, especially in those with moderte to severe depression. Balance impairments are common in individuals with schizophrenia, increasing the risk of falls and functional limitations. Aerobic exercise positively influences motor function, including balance control^70^. It improves balance by strengthening key muscles in the legs, hips, and core. Additionally, regular aerobic training promotes neuroplastic changes in the central nervous system, enhancing sensorimotor integration, proprioception, and coordination^71^.

This study contributes to the limited but growing body of literature supporting aerobic training as an effective intervention for improving mobility in individuals with schizophrenia^72–74^. Schizophrenia is frequently associated with motor disturbances such as impaired gait, reduced muscle strength, and decreased physical fitness, which collectively diminish functional independence, and quality of life^74^. These impairments often emerge early and persist throughout the illness, exacerbating sedentary behavior and increasing the risk of comorbid physical conditions. Regular aerobic exercise has been shown to improve key components of physical functioning, including cardiorespiratory fitness, muscular endurance, balance, and coordination, all of which are essential for enhancing mobility and daily functioning in this population^74,75^. Beyond physical benefits, aerobic training also exerts neurobiological effects that may further support motor and cognitive improvements. Research indicates that aerobic exercise promotes neuroplasticity, and neuroprotection within the central nervous system by enhancing cerebral blood flow, stimulating neurogenesis, and supporting synaptic connectivity^72–75^. These mechanisms contribute to improved movement control and psychomotor functioning, making aerobic training a valuable adjunct to conventional treatment approaches.

This study demonstrates that the integration of a cognitive, and music intervention, along with aerobic training yields cumulative benefits. It confirms that cognitive interventions are a critical component of any multimodal intervention aimed at improving depression, balance, and mobility in patients with schizophrenia and severe depression. The combination of these therapies offers a multifaceted and holistic approach to alleviating depression, addressing cognitive, emotional, and physical dimensions simultaneously. Research on combined treatments has shown significant reductions in depressive symptoms and improvements in overall functioning and well-being, both as individual therapies and in the combination of cognitive and aerobic exercise treatment^76^. Balance impairments are common in schizophrenia, increasing the risk of falls and functional limitations. This study demonstrated the synergistic benefits of combining cognitive, music, and aerobic exercise therapies by addressing various aspects of balance impairment concurrently. Research on individual and combined treatments, such as a cognitive intervention, and aerobic exercise^77^, have shown significant improvements in balance measures, surpassing the results achieved with cognitive interventions or aerobic training in isolation^77^. While cognitive, music interventions, and aerobic training have been explored as potential interventions for improving functioning in individuals with schizophrenia, this study shows that combining these approaches is a promising comprehensive treatment strategy to enhance mobility. Furthermore, it demonstrates that a multimodal therapeutic intervention is superior to single- or dual-modality interventions. The TUG test, commonly used to assess mobility and functional ability in various populations, including those with schizophrenia, supports this finding. Although previous research have investigated the effects of cognitive^78^, music^79^, and aerobic training^80^ on mobility impairments, this study uniquely confirms the individual positive outcomes and highlights the significant improvements achieved through various dual-modalities and ultimately as a triple-modality.

This study has several limitations to consider in that it exclusively involved a male population, which may limit generalisability and overlook gender-specific phenomena. Additionally, participants with schizophrenia were on personalised medication regimens, which may have varied in type, dosage, and timing, though this was not assessed. The absence of a non-treatment control group could also be viewed as a limitation. However, the study used a randomised active-controlled trial in accordance with the World Medical Association’s Declaration of Helsinki, allowing for the comparative evaluation of each trial arm against the best proven intervention(s), specifically the cognitive intervention or treatment-as-usual. This approach addressed a comparative effectiveness research question rather than evaluating outcomes in a single treatment strategy cohort. Another potential limitation of this study is the absence of psychosis-related outcome measures. This is because the study focused on addressing key aspects of schizophrenia that significantly impact daily functioning, such as depression, balance, and mobility, rather than directly measuring psychotic symptoms. This decision was based on several factors. While psychosis is a core feature of schizophrenia, antipsychotic medications remain the standard treatment for managing these symptoms, and all participants were on stable medication regimens. Therefore, the study aimed to explore complementary interventions that could enhance well-being beyond pharmacological treatment. In addition, assessing psychotic symptoms reliably requires structured clinical interviews, which can be influenced by daily fluctuations, medication effects, and interviewer bias, making it challenging to measure consistent outcomes. Instead, the study employed validated, objective tools to evaluate depression, motor function, and mobility, areas that are often neglected but have a profound effect on quality of life. Additionally, cognitive intervention, one of the interventions studied, has been shown to indirectly benefit cognitive deficits associated with psychosis, such as executive dysfunction and attention impairments. Although music was incorporated as a music-based activity intervention, it was not delivered within a structured music therapy framework led by a credentialed music therapist. A further limitation of this study is the lack of equivalence in therapeutic intent across conditions in that while TAU included access to psychotherapy, the aerobic and music-based interventions were structured as physical and recreational activities rather than therapies specifically targeting psychological symptoms, thereby limiting the comparability of depression outcomes across groups.

Given these considerations, the study prioritised practical and meaningful outcome measures while acknowledging that future research could further explore whether these interventions also have secondary effects on psychotic symptoms. A notable strength was the use of an inpatient population, which enabled control over diet, activity, and adherence to treatment conditions. While aerobic exercise was implemented in this study as a physical activity intervention rather than a form of psychotherapy, future research should explore the potential of physical activity (i.e., dance/movement therapy), which integrates structured physical movement with a psychotherapeutic framework, as a complementary or alternative intervention for individuals with schizophrenia, particularly given emerging evidence supporting its efficacy in this population. While the interventions in this study differ in their therapeutic scope, future research should consider evaluating outcomes across interventions with equivalent therapeutic frameworks (i.e., cognitive therapy, dance/movement therapy, and music therapy) to more accurately assess differential effects on psychological and functional outcomes in individuals with schizophrenia.

## Conclusions

This single-blind randomised active-controlled trial investigated the effects of various treatment modalities on depression, balance, and mobility in male patients with schizophrenia, even in those with severe depression. Our findings revealed significant improvements across all treatment groups, highlighting the efficacy of aerobic exercise, and cognitive, and music interventions in addressing both psychological and physical symptoms associated with schizophrenia and severe depression. Notably, the multimodal approach, which combined these three therapies, showed superior outcomes compared to single or dual-modality interventions, with the comprehensive triple-modal intervention being the most effective for improving depression, balance, and mobility. These results emphasise the need for a holistic and scalable treatment model for schizophrenia to optimise patient outcomes. Furthermore, integrating diverse therapeutic modalities may enhance treatment engagement and adherence, allowing patients to explore multiple avenues for symptom relief tailored to their preferences and needs.

## Data Availability

The datasets used and/or analysed during the current study are available from the corresponding author on reasonable request.

## References

[1] Noorbala, A. A. et al. Mental health survey of the Iranian adult population in 2015. *Arch. Iran. Med.* **20**, 128–134 (2017). 28287805

[2] National Institute for Health and Care Excellence (NICE). *Psychosis and schizophrenia: How common are psychotic disorders?* (2021). https://cks.nice.org.uk/topics/psychosis-schizophrenia/background-information/prevalence/

[3] Möller, H., Hasan, A. & Falkai, P. World federation of societies of biological psychiatry (WFSBP) guidelines for biological treatment of schizophrenia. *World J. Biol. Psychiatry*. **16** (3), 142–170 (2015). 25822804 10.3109/15622975.2015.1009163

[4] Birchwood, M., Iqbal, Z. & Upthegrove, R. Psychological pathways to depression in schizophrenia: studies in acute psychosis, post psychotic depression and auditory hallucinations. *Eur. Arch. Psychiatry Clin. Neurosci.* **255**, 202–212 (2005). 15995904 10.1007/s00406-005-0588-4

[5] Conley, R. R. et al. The burden of depressive symptoms in the long-term treatment of patients with schizophrenia. *Schizophr Res.* **90** (1–3), 186–197 (2007). 17110087 10.1016/j.schres.2006.09.027 PMC1937504

[6] Brochet, B. & Ruet, A. Cognitive impairment in multiple sclerosis with regards to disease duration and clinical phenotypes. *Front. Neurol.* **10**, 261 (2019). 30949122 10.3389/fneur.2019.00261 PMC6435517

[7] Harvey, P. D. Cognitive functioning and disability in schizophrenia. *Curr. Dir. Psychol. Sci.* **19**, 249–254 (2010).

[8] Molnar, M. J. et al. Early-onset schizophrenia with predominantly negative symptoms: a case study of a drug-naive female patient treated with Cariprazine. *Front. Pharmacol.* **11**, 477 (2020). 32390838 10.3389/fphar.2020.00477 PMC7191004

[9] Apthorp, D. et al. Postural sway abnormalities in schizotypal personality disorder. *Schizophr Bull.* **45** (3), 512–521 (2019). 30376125 10.1093/schbul/sby141 PMC6483590

[10] Das, S. et al. An exploratory study from Eastern India on neurological soft signs and spontaneous movement disorders in schizophrenia spectrum disorders. *Open. J. Psychiatry Allied Sci.* **10** (1), 3 (2019). 30868105 10.5958/2394-2061.2019.00001.6 PMC6411054

[11] Drevets, W. C. & Furey, M. L. Replication of scopolamine’s antidepressant efficacy in major depressive disorder: a randomized, placebo-controlled clinical trial. *Biol. Psychiatry*. **67** (5), 432–438 (2010). 20074703 10.1016/j.biopsych.2009.11.021 PMC3264395

[12] Kirkpatrick, B. et al. Is schizophrenia a syndrome of accelerated aging? *Schizophr Bull.* **34** (6), 1024–1032 (2008). 18156637 10.1093/schbul/sbm140 PMC2632500

[13] Tsai, M. T. et al. Association between frailty and its individual components with the risk of falls in patients with schizophrenia spectrum disorders. *Schizophr Res.* **197**, 138–143 (2018). 29395605 10.1016/j.schres.2018.01.023

[14] Stubbs, B. et al. Predictors of falls and fractures leading to hospitalization in people with schizophrenia spectrum disorder: A large representative cohort study. *Schizophr Res.* **201**, 70–78 (2018). 29793816 10.1016/j.schres.2018.05.010

[15] Helfer, B. et al. Efficacy and safety of antidepressants added to antipsychotics for schizophrenia: a systematic review and meta-analysis. *Am. J. Psychiatry*. **173** (9), 876–886 (2016). 27282362 10.1176/appi.ajp.2016.15081035

[16] Sukhato, K. et al. Efficacy of home-based non-pharmacological interventions for treating depression: a systematic review and network meta-analysis of randomised controlled trials. *BMJ Open.* **7**(7), e014499 (2017). 10.1136/bmjopen-2016-014499 PMC5734422 28706086

[17] Shaw, I., Cronje, M. & Shaw, B. S. Group-based exercise as a therapeutic strategy for the improvement of mental outcomes in mild to moderate alzheimer’s patients in low resource care facilities. *Asian J. Sports Med.* **12** (1), e106593 (2021).

[18] Ward, N. et al. Enhanced learning through multimodal training: evidence from a comprehensive cognitive, physical fitness, and neuroscience intervention. *Sci. Rep.* **7** (1), 5808 (2017). 28724914 10.1038/s41598-017-06237-5 PMC5517605

[19] Sangelaji, B. et al. Interference effect of prior explicit information on motor sequence learning in relapsing-remitting multiple sclerosis patients. *Malays J. Med. Sci.* **24**, 69–80 (2017). 28381930 10.21315/mjms2017.24.1.8 PMC5346005

[20] Wojtalik, J. A. et al. Confirmatory efficacy of cognitive enhancement therapy for early schizophrenia: results from a multisite randomized trial. *Psychiatr Serv.* **73** (5), 501–509 (2022). 34470506 10.1176/appi.ps.202000552 PMC8888780

[21] Ding, J. et al. Effectiveness and safety of music-supported therapy on mood in post-stroke rehabilitation patients: a protocol for systematic review and meta-analysis. *Med* **100**(12), e25077 (2021). 10.1097/MD.0000000000025077 PMC9282033 33761665

[22] Shahpouri, M. M. et al. Evaluation of cognitive rehabilitation on the cognitive performance in multiple sclerosis: A randomized controlled trial. *J. Res. Med. Sci.* **24**, 110 (2019). 31949461 10.4103/jrms.JRMS_124_19 PMC6950338

[23] Swain, R. et al. On aerobic exercise and behavioral and neural plasticity. *Brain Sci.* **2**, 709–744 (2012). 24961267 10.3390/brainsci2040709 PMC4061809

[24] Ivelize, F. & Adérito, S. Effectiveness of a sensorimotor exercise program on proprioception, balance, muscle strength, functional mobility and risk of falls in older people. *Front. Physiol.* **15**, 1309161 (2024). 38694207 10.3389/fphys.2024.1309161 PMC11061438

[25] Groves, S. J. et al. Changes in neuropsychological function after treatment with metacognitive therapy or cognitive behavior therapy for depression. *Depress. Anxiety*. **32**, 437–444 (2015). 25677736 10.1002/da.22341

[26] Yıldırım, M. et al. Postural control and executive functioning in patients with schizophrenia. *Eur. Psychiatry*. **41**, S195–S195 (2017).

[27] Hou, X. The application of music therapy in autism spectrum disorder, depression, and anxiety: effects on symptom relief, social skill enhancement, and emotional regulation. *Adv. Humanit. Res.* **10**, 23–27 (2024).

[28] Bella, S. D. et al. Gait improvement via rhythmic stimulation in parkinson’s disease is linked to rhythmic skills. *Sci. Rep.* **7**, 42005 (2017). 28233776 10.1038/srep42005 PMC5324039

[29] Benoit, C. S. et al. Musically cued gait-training improves both perceptual and motor timing in parkinson’s disease. *Front. Hum. Neurosci.* **8** (494), 1–11 (2014). 25071522 10.3389/fnhum.2014.00494 PMC4083221

[30] Khanmohammadi, R. et al. Effect of cognitive and exercise rehabilitation on gait in male schizophrenic patients suffering from depression disorder. *Russ Open. Med. J.* **9** (4), 409 (2020).

[31] Geretsegger, M. et al. Music therapy for people with schizophrenia and schizophrenia-like disorders. *Cochrane Database Syst. Rev.* **5** (5), CD004025 (2017). 28553702 10.1002/14651858.CD004025.pub4 PMC6481900

[32] Strassnig, M. et al. Physical performance and disability in schizophrenia. *Schizophr Res. Cogn.* **2**, 112–121 (2014). 10.1016/j.scog.2014.06.002 PMC4171045 25254158

[33] Ellis, N., Crone, D., Davey, R. & Grogan, S. Exercise interventions as an adjunct therapy for psychosis: a critical review. *Br. J. Clin. Psychol.* **46** (Pt 1), 95–111 (2007). 17472204 10.1348/014466506x122995

[34] Bademli, K., Lök, N. & Lök, S. The effect of a physical activity intervention on burden and depressive symptoms in depressed family caregivers of patients with schizophrenia: a randomized controlled trial. *J. Phys. Act. Health*. **20** (12), 1109–1115 (2023). 37633655 10.1123/jpah.2022-0335

[35] Kuo, Y. C., Chang, D. Y. & Liao, Y. H. Twelve-weeks of bench-step exercise training ameliorates cardiopulmonary fitness and mood state in patients with schizophrenia: a pilot study. *Medicina* **57** (2), 149 (2021). 33562247 10.3390/medicina57020149 PMC7915556

[36] Huang, Y. et al. Effects of aerobic walking on cognitive function in patients with schizophrenia: A randomized controlled trial. *J. Psychiatr Res.* **134**, 173–180 (2012). 10.1016/j.jpsychires.2020.12.062 33388700

[37] Ovosi, J. O., Ibrahim, M. S. & Bello-Ovosi, B. O. Randomized controlled trials: ethical and scientific issues in the choice of placebo or active control. *Ann. Afr. Med.* **16** (3), 97–100 (2017). 28671148 10.4103/aam.aam_211_16 PMC5579903

[38] World Medical Association. *WMA Declaration of Helsinki-ethical principles for medical research involving human subjects. World Health Organisation*. (2013). https://www.who.int/bulletin/volumes/79/4/373.pdf 10.1001/jama.2013.281053 24141714

[39] Furlanetto, L. M., Mendlowicz, M. V. & Bueno, J. R. The validity of the Beck depression Inventory-Short form as a screening and diagnostic instrument for moderate and severe depression in medical inpatients. *J. Affect. Disord*. **86** (1), 87–91 (2005). 15820275 10.1016/j.jad.2004.12.011

[40] Hedayati, S. et al. Validation of depression screening scales in patients with CKD. *Am. J. Kidney Dis.* **54**(3):433–439 . 10.1053/j.ajkd.2009.03.016 PMC3217720 19493600

[41] Wang, Y. & Gorenstein, C. The Beck depression inventory: uses and applications in The Neuroscience of Depression (eds Martin, C. R. et al.) 165–174 (Academic, (2021).

[42] Johnson, B. L. & Nelson, J. K. *Practical Measurements for Evaluation in Physical Education* 4th edn (Burgess, 1979).

[43] Podsiadlo, D. & Richardson, S. The timed up & go: a test of basic functional mobility for frail elderly persons. *J. Am. Geriatr. Soc.* **39** (2), 142–148 (1991). 1991946 10.1111/j.1532-5415.1991.tb01616.x

[44] Nejati, V., Pouretemad, H. R. & Bahrami, H. Attention training in rehabilitation of children with developmental stuttering. *NeuroRehabilitation* **32** (2), 297–303 (2013). 23535791 10.3233/NRE-130847

[45] Haskell, W. L. Guidelines for physical activity and health in the united states: evolution over 50 years. *ACSMs Health Fit. J.* **23** (5), 5–8 (2019).

[46] Reschke-Hernández, A. E. The clinical practice model for persons with dementia: application to music therapy. *J. Music Ther.* **39** (2), 133–141 (2021).

[47] Guetin, S. et al. Effect of music therapy on anxiety and depression in patients with alzheimer’s type dementia: randomized, controlled study. *Dement. Geriatr. Cogn. Disord*. **28** (1), 36–46 (2009). 19628939 10.1159/000229024

[48] Beck, A. T. & Rector, N. A. Cognitive therapy of schizophrenia: a new therapy for the new millennium. *Am. J. Psychother.* **54** (3), 291–300 (2000). 11008627 10.1176/appi.psychotherapy.2000.54.3.291

[49] Joung, H. J. & Lee, Y. Effect of creative dance on fitness, functional balance, and mobility control in the elderly. *Gerontol* **65** (5), 537–546 (2019). 10.1159/000499402 31055579

[50] John, A. et al. Effectiveness of IADL interventions to improve functioning in persons with schizophrenia: A systematic review. *Int. J. Soc. Psychiatry*. **68** (3), 500–513 (2022). 34802260 10.1177/00207640211060696

[51] Hoffman, G. J. et al. Depressive symptomatology and fall risk among community-dwelling older adults. *Soc. Sci. Med.* **178**, 206–213 (2017). 28279573 10.1016/j.socscimed.2017.02.020 PMC5411980

[52] Jia, R. et al. The effectiveness of adjunct music therapy for patients with schizophrenia: a meta-analysis. *Psychiatry Res.* **289**, 112983 (2020). 33002835 10.1016/j.psychres.2020.113464

[53] Talwar, N. et al. Music therapy for in-patients with schizophrenia: exploratory randomised controlled trial. *Br. J. Psychiatry*. **189** (5), 405–409 (2006). 17077429 10.1192/bjp.bp.105.015073

[54] Carrick, F. R., Oggero, E. & Pagnacco, G. Posturographic changes associated with music listening. *J. Altern. Complement. Med.* **13** (5), 519–526 (2007). 17604555 10.1089/acm.2007.7020

[55] Sijobert, B. et al. Interest of the task of stepping in place in the assessment of freezing in parkinson’s disease patients. *Ann. Phys. Rehabil Med.* **58** (1), e71–e72 (2015).

[56] Pavlicevic, M., Trevarthen, C. & Duncan, J. Improvisational music therapy and the rehabilitation of persons suffering from chronic schizophrenia. *J. Music Ther.* **31** (2), 86–104 (1994).

[57] Mendlowicz, M. V. et al. A comparison of descriptive characteristics of male outpatients and inpatients with affective disorders. *Int. Clin. Psychopharmacol.* **13** (6), 245–252 (1998). 9861574 10.1097/00004850-199811000-00002

[58] Harvey, P. D., Twamley, E. W., Pinkham, A. E., Depp, C. A., Patterson, T. L. & Depression in schizophrenia: associations with cognition, functional capacity, everyday functioning, and self-assessment. *Schizophr Bull.* **43** (3), 575–582 (2017). 27440672 10.1093/schbul/sbw103 PMC5463852

[59] Kramer, M. S. et al. Antidepressants in ‘Depressed’ Schizophrenic Inpatients. *Med*. (1989). https://www.semanticscholar.org/paper/Antidepressants-in-‘Depressed’-Schizophrenic-Kramer-Vogel/c05277e1a7bf09066306007d49c5f449da713db7 10.1001/archpsyc.1989.01810100064012 2679483

[60] Barnes, T. R. E., Curson, D. A., Liddle, P. F. & Patel, M. The nature and prevalence of depression in chronic schizophrenic in-patients. *Br. J. Psychiatry*. **154** (4), 486–491 (1989). 2574068 10.1192/bjp.154.4.486

[61] Heald, A. H., Morris, J. & Soni, S. D. Characterisation of depression in patients with schizophrenia. *Indian J. Med. Res.* **127**, 544–550 (2008). 18765872

[62] Scholes, B. & Martin, C. R. Measuring depression in schizophrenia with questionnaires. *J. Psychiatr Ment Health Nurs.* **20** (1), 17–22 (2012). 22340193 10.1111/j.1365-2850.2012.01877.x

[63] Chien, T., Chern, J., Wang, S. & Yang, T. Effects of multitask training on cognition and motor control in people with schizophrenia spectrum disorders. *Plos One* **17**(6), e0264745 (2022). 10.1371/journal.pone.0264745 PMC9246115 35771832

[64] Feldman, R., Schreiber, S., Pick, C. G. & Been, E. Gait, balance and posture in major mental illnesses: depression, anxiety and schizophrenia. *Austin Med. Sci.* **5** (1), 1039 (2020).

[65] Braun Janzen, T., Haase, M. & Thaut, M. H. Rhythmic priming across effector systems: a randomized controlled trial with parkinson’s disease patients. *Hum. Mov. Sci.* **64**, 355–365 (2019). 30852469 10.1016/j.humov.2019.03.001

[66] Odufuwa-Pelote, A., Johnson, K., Jones, S. & Carter, H. Effects of music therapy in reducing falls among patients with dementia in a long-term facility: a cohort study. *Nurs Resid. Care* **26**(4) (2024).

[67] Chatterjee, D., Hegde, S. & Thaut, M. Neural plasticity: the substratum of music-based interventions in neurorehabilitation. *NeuroRehabilitation* **48** (2), 155–166 (2021). 33579881 10.3233/NRE-208011 PMC7613141

[68] Segev-Jacubovski, O. et al. The interplay between gait, falls and cognition: can cognitive therapy reduce fall risk? *Expert Rev. Neurother.* **11** (7), 1057–1075 (2011). 21721921 10.1586/ern.11.69 PMC3163836

[69] Bhattacharya, P., Chatterjee, S. & Roy, D. Impact of exercise on brain neurochemicals: a comprehensive review. *Sport Sci. Health*. **1**, 48 (2023).

[70] Jafari, R. & Bafandeh, H. The effectiveness of cognitive rehabilitation on the improvement of depression symptoms and brain wave pattern in patients with depression disorder. *J. Community Health*. **13** (3), 64–72 (2019).

[71] Zech, A. et al. Balance training for neuromuscular control and performance enhancement: a systematic review. *J. Athl Train.* **45** (4), 392–403 (2010). 20617915 10.4085/1062-6050-45.4.392 PMC2902034

[72] Maurus, I. et al. Neurobiological effects of aerobic exercise, with a focus on patients with schizophrenia. *Eur. Arch. Psychiatry Clin. Neurosci.* **269**, 499–515 (2019). 31115660 10.1007/s00406-019-01025-w

[73] Cuoco, F. et al. Get up! Functional mobility and metabolic syndrome in chronic schizophrenia: effects on cognition and quality of life. *Schizophr Res. Cogn.* **28**, 100245 (2022). 35251942 10.1016/j.scog.2022.100245 PMC8892146

[74] Heggelund, J. et al. Therapeutic effects of maximal strength training on walking efficiency in patients with schizophrenia – a pilot study. *BMC Res. Notes*. **5**, 344 (2012). 22759719 10.1186/1756-0500-5-344 PMC3568714

[75] Gilbert, C., Earleywine, M. & Altman, B. R. Perceptions of cognitive behavioral therapy, aerobic exercise, and their combination for depression. *Prof. Psychol. Res. Pr*. **52** (6), 551 (2021). 10.1080/07448481.2023.2185461 36862695

[76] Amorós-Aguilar, L., Rodríguez-Quiroga, E., Sánchez-Santolaya, S. & Coll-Andreu, M. Effects of combined interventions with aerobic physical exercise and cognitive training on cognitive function in stroke patients: a systematic review. *Brain Sci.* **11** (4), 473 (2021). 33917909 10.3390/brainsci11040473 PMC8068294

[77] Pearsall, R. & Smith, D. M. Psychological and psychophysiological considerations in physical activity, exercise, and sports performance in *Handbook of Sport Psychology* (3rd ed.) (eds. Tenenbaum, G., Eklund, R.C. & Kamata, A) 287–309 (John Wiley & Sons, Inc., (2011).

[78] Silverman, M. J. et al. Music therapy for mental health and substance abuse disorders in Introduction To Music Therapy Practice. (eds Hall, D. E., Hall, S. J. & Ramos, W. A.) 293–316 (Routledge, (2019).

[79] Firth, J. et al. A systematic review and meta-analysis of exercise interventions in schizophrenia patients. *Psychol. Med.* **47** (16), 2741–2755 (2017). 10.1017/S0033291714003110 25650668

[80] Paulus, J. K. et al. Opportunities and challenges in using studies without a control group in comparative effectiveness reviews. *Res. Syn Meth*. **5**, 152–161 (2014). 10.1002/jrsm.1101 26052654

